# An immune infiltration-related prognostic model of kidney renal clear cell carcinoma with two valuable markers: CAPN12 and MSC

**DOI:** 10.3389/fonc.2023.1161666

**Published:** 2023-03-21

**Authors:** Guang Xia, Song Wu, Xiaoyu Cui

**Affiliations:** ^1^ Department of Orthopaedics of the 3rd Xiangya Hospital, Central South University, Changsha, China; ^2^ Department of Anesthesiology of the 3rd Xiangya Hospital, Central South University, Changsha, China

**Keywords:** ccRCC, prognostic model, immune infiltration, somatic mutation, cell proliferation

## Abstract

**Background:**

Since its discovery, clear cell renal cell carcinoma (ccRCC) has been the most prevalent and lethal kidney malignancy. Our research aims to identify possible prognostic genes of ccRCC and to develop efficient prognostic models for ccRCC patients based on multi-omics investigations to shed light on the treatment and prognosis of ccRCC.

**Methods:**

To determine a risk score for each patient, we screened out differentially expressed genes using data from tumor samples, and control samples mined from The Cancer Genome Atlas (TCGA) and GTEx datasets. Somatic mutation and copy number variation profiles were analyzed to look for specific genomic changes connected to risk scores. To investigate potential functional relationships of prognostic genes, gene set variation analysis (GSVA) and gene set enrichment analysis (GSEA) were carried out. We created a prognostic model by fusing risk ratings with other clinical variables. For validation, the 786-O cell line was used to carry out the dual-gRNA approach to knock down CAPN12 and MSC. This was followed by qRT-PCR to verify the knockdown of CAPN12 and MSC.

**Results:**

For ccRCC, seven predictive genes were discovered: PVT1, MSC, ALDH6A1, TRIB3, QRFPR, CYS1, and CAPN12. The most enriched pathways in the GSVA study and GSEA analysis promote tumorigenesis and immune system modulation. The risk score derived from prognostic genes corresponds with immune infiltration cells and helps predict how well a medicine will work. The mutation of numerous oncogenes was also linked to a high-risk score. A prognostic model with a high ROC value was created for the risk score. An *in vitro* study demonstrates that the suppression of CAPN12 and MSC dramatically reduced the ability of 786-O cells to proliferate in the CCK-8 proliferation assay and plate clonality assays.

**Conclusions:**

A thorough prognostic model with good performance has been developed for ccRCC patients using seven prognostic genes that were discovered to be related to ccRCC prognosis. In ccRCC, CAPN12 and MSC were significant indicators and would make good therapeutic targets.

## Background

Kidney cancer has long been a common malignant tumor in the urinary system, with an increasing incidence rate worldwide. In the USA, 65,000 individuals are newly diagnosed with kidney cancers per year (1). Among all kinds, clear cell renal cell carcinoma (ccRCC) accounts for approximately 80% of kidney cancers, which also correlates with worse survival outcomes (2). Although the 5-year overall survival (OS) of patients with early diagnosis of ccRCC is about 90%, the 5-year OS for patients diagnosed at an advanced stage is down to 12% (3). Unfortunately, almost 20% of cases are in advanced malignant stages when diagnosed (4). Regarding treatment, nephrectomy continues to be the optimal approach for localized ccRCC. A phase 3 clinical trial has proved that nephrectomy with adjuvant chemotherapy increased the progressive free survival (PFS) of ccRCC patients to 6.8 years compared with nephrectomy alone (5.8 years) (5). Although chemotherapy is a good option for multiple cancer types, ccRCC shows resistance to chemotherapy *via* secreting vascular endothelial cell growth factor (VEGF) (6). Other molecules, such as the mammalian target of rapamycin (mTOR) and the mitogen-activated protein kinase (MAPK), have also been demonstrated to be involved in the carcinogenesis of ccRCC and dampen the effectiveness of chemotherapy (7, 8).

Recently, immunotherapies combined with conventional surgical resection and radiotherapy have gradually improved the clinical management of ccRCC (9). However, the mortality rate of ccRCC remains high due to diagnostic difficulty at the early stage of the disease. Thus 30% of patients inevitably would suffer from tumor recurrence and progression (9). Combining ccRCC prognostic genes, researchers have built some predictive models for ccRCC patients based on online databases, such as The Cancer Genome Atlas (TCGA), with many genetic ccRCC samples. However, no prognostic model of ccRCC has been widely accepted. Thus, a risk stratification model identifying ccRCC-related biomarkers and assessing the prognosis of ccRCC patients is urgently needed. In this study, we present a ccRCC prognostic model after mining and screening multiple predictive genes from the TCGA dataset, aiming to shed light on optimizing the clinical management of ccRCC patients.

## Materials and methods

### Datasets and preprocessing

We gathered two cohorts of patients with ccRCC for this study: GSE29609 (microarray) from the platform (GPL1708) and TCGA Kidney Renal Clear Cell Carcinoma (KIRC) (RNA-seq) cohort. Raw data from the microarray dataset generated by Agilent was downloaded from the Gene Expression Omnibus (GEO) (https://www.ncbi.nlm.nih.gov/geo/). Gene expression profile induced by Illumina and corresponding clinical information were downloaded from The Cancer Genome Atlas (TCGA) data source (https://xena.ucsc.edu). Raw data for the dataset from Agilent were processed using the RMA algorithm for background adjustment in the *limma* software package. The raw data from Illumina was processed using the lumi software package (10). For the TCGA cohort, RNA-sequencing data (FPKM values) were transformed into transcripts per kilobase million (TPM) values that are more similar to the values from the microarray. Samples without survival information were eliminated, 528 KIRC samples in TCGA were screened out for the risk score construction, and 39 KIRC samples in GEO were screened out for external validation of the risk score. One hundred standard pieces were downloaded from https://xenabrowser.net/datapages/?cohort=TCGA%20TARGET%20GTEx&removeHub=https%3A%2F%2Fxena.treehouse.gi.ucsc.edu%3A443, among which 28 regular renal models were from the GTEx database (https://xena.ucsc.edu), and 72 normal renal samples were from the TCGA database (https://xena.ucsc.edu). These 100 normal samples were already combined, so removing the batch effect was unnecessary. The TCGA KIRC cohort was randomly divided into two equal parts: the train set (set 1) and the validation set (set 2). The total TCGA KIRC data were used as another verification set (set 3), while the GEO cohort was used as the external validation set in the following studies (set 4).

### Identification of differentially expressed genes in KIRC

Probes without corresponding gene symbols were filtered out, and the average value of gene symbols with multiple searches was calculated. Between the two groups, the Linear Models for Microarray Data Analysis (limma) package (10) was used to screen the differentially expressed genes (DEGs). Threshold values were set as adjusted P<0.05 and the absolute value of logFC> 2. A principal component analysis was also applied to categorize the data further to assess the DEGs’ accuracy.

### Screening and confirmation of the prognostic value of the genes

By intersecting the obtained differential expressed gene with the genes of TCGA, genes for further analysis were obtained. In the training set (set 1), univariate Cox proportional hazard regression analysis was performed using the survival package in R to investigate the relationship between patients’ overall survival (OS) and gene expression level. Genes were considered significant with prognostic potential at a P-value<0.05. Next, we applied an L1-penalized (Lasso) regression to identify the differentially expressed genes with independent predictive values. Lasso regression is a valuable method to determine interpretable prediction rules in high dimension data (11). We obtained a set of prognostic genes and their corresponding LASSO coefficients based on the highest lambda value selected through 1,000 cross-validations in the Lasso method (lambda.1se). To evaluate whether the selected genes were related to the prognosis of KIRC patients, patients of set 1 were assigned into two groups based on the median expression value of each gene. Kaplan-Meier plots were used to determine their prognostic value, and P<0.05 was considered statistically significant. A genes-based survival risk assessment model was established using the LASSO coefficients. Then, patients were divided into low-risk and high-risk groups using the median risk scores in the other three sets. Kaplan-Meier plots and Log-rank tests were used to estimate and compare the OS of patients between the two risk groups; P<0.05 was set as the cutoff. The time-dependent receiver operating characteristic (ROC) curve and the area under the curve (AUC) were applied to evaluate the prediction accuracy of the risk model and the selected genes. Furthermore, stratified survival analyses were also conducted to explore whether the gene-based risk assessment model has predictive value among different age groups (older or younger than 60), primary tumor lesions (T1, T2, T3, T4), and stage (stage I, stage ii, stage iii, stage iv).

### Consensus clustering of prognostic genes

To investigate the function of seven prognostic genes in KIRC, we clustered the KIRCs into different groups with “ConsensusClusterPlus” (50 iterations, resample rate of 80%, and Pearson correlation). PCA with the R package for R v3.4.1 was adopted to study the gene expression patterns in different KIRC groups.

### Genomic alterations of samples clustered by risk scores

To determine whether risk score levels are associated with specific genomic characteristics in ccRCC, we performed copy number variation (CNV) and somatic mutation analysis using the TCGA dataset. GSITIC analysis was adopted to determine the genomic event enrichment.

### Prognostic model based on clinical features and risk score

Univariate Cox proportional hazard regression analysis was performed using the survival package for the risk score and clinical features (Age, Tumor primary lesion, Stage) with a P value <0.05 as the cutoff. Then we built a Multivariate Cox model based on the selected features, and the Nomogram chart was drawn using the replot package. The Calibration curve and the AUC assessed the risk model.

### Gene set variation analysis and geneset enrichment analysis

The gene set *variation analysis (GSVA) and geneset enrichment analysis (GSEA)* packages were used to calculate the enrichment status in Gene Ontology (GO) and Kyoto Encyclopedia of Genes and Genomes (KEGG) terms of TCGA samples. Correlation analysis was performed by expression values of risk score, GO terms, and KEGG terms. The items with p<0.05 and a high correlation coefficient were selected (12).

### Immunological function analyses

A single sample gene set enrichment analysis (ssGSEA) was performed using R software to quantify 28 tumor-infiltrating immune cells (Foroutan et al., 2018). Correlation analysis between risk score and tumor-infiltrating immune cell expressions was performed using gene expression profiles from the TCGA datasets.

### Prediction of chemotherapeutic and immunotherapy response

The Tumor Immune Dysfunction and Exclusion (TIDE) algorithm was performed to infer individual responses to immunotherapy, such as immune checkpoint blockade (e.g., anti-PD-1 therapy). The submap analysis was applied to show the difference in response to anti-PD-1 and CTAL-4 therapy (13). The chemotherapeutic response for each ccRCC patient was predicted according to the public pharmacogenomic database, Genomics of Drug Sensitivity in Cancer (GDSC, www.cancerrxgene.org). The prediction of drug sensitivity (IC50) values was conducted using the R package “prophetic” (14).

### CAPN12 and MSC knockdown

Knockdown plasmids were constructed by the dual-gRNA method (15), targeting CAPN12 and MSC. Vectors without specific gRNAs were used as control. All PCR products were verified by DNA sequencing. Transfection of plasmids was carried out using Lipofectamine 2000 (Invitrogen, USA) according to the manufacturer’s instructions. After the transfection, cells were seeded and grown in the RPMI-1640 supplemented with 5% FBS. Then 786-O cell clones were picked, and the expression of CAPN12 and MSC were validated by qRT-PCR. Plate clonality assays were also used to measure the impact of knockdown on cell clonality and cell cycle in the 786-O cell line after silencing CAPN12 and MSC.

### Quantitative real-time polymerase chain reaction

Three biological replicates were analyzed, with technical replicates for each triplicate biological sample. Total RNAs were extracted, reversed, and transcribed into cDNA by HiScript Q RT SuperMix for qRT-PCR. ChamQ SYBR qRT-PCR Master Mix was used for qRT-PCR experiments, and its protocol was as follows: 95°C 30 s, 95°C 10 s, 60°C 30 s, for a total of 40 cycles reactions. The expression level of target genes was quantified using the 2-ΔΔCT method. GADPH was used as the internal standard. The primers are as follows: CAPN12, 5’-CTCCATTTCGACACCGTGCAG-3’, 5’-GAGTTGAAGCCACGCACCCA-3’; MSC, 5’-CAACTCGTAGTCCACGCTCC-’3, 5’-TAAAAACCCAGGCCGGGAAG-3’.

### Cell proliferation assay

Cell Counting Kit-8 (CCK-8) proliferation assay was conducted to assess the proliferation ability of cells according to the manufacturer’s instructions. After cell counting, 1×104 cells were seeded into 96-well plates and incubated at 37°C for 24 h, 48 h, and 72 h. ten μL CCK-8 reagent was added into each well, and the absorbance at 450 nm was tested one h later.

### Colony forming assay

Cells were digested and plated in 6-well plates (300 cells per well) and cultured with 5% CO2 at 37C for two weeks. The colonies were then fixed with 4% methanol (1 ml per well) for 15 minutes and stained with crystal violet for 30 minutes at room temperature. After the photograph, discoloration was performed with 10% acetic acid, and cells were measured absorbance at 550 nm.

### Statistical analysis

All statistical analyses were performed using R software. A two‐tailed t-test and one‐way ANOVA determined significant quantitative differences between and among groups. The chi-square test was used to analyze the correlation of the classified data. The Kaplan–Meier method calculated the overall survival difference. Cox regression analysis was performed using the survival package in R. Spearman correlation to measure the strength of the association between two ranked variables. The *gene sets enrichment analysis* (*GSVA*) box was used to calculate the enrichment status in GO (Biological Process) (12). The R package survival ROC was used to plot and visualize receiver operating characteristic (ROC) curves to calculate the area under the curve (AUC) (16). All figures and statistical analyses were performed based on R language for Windows, version 3.5.1(http://www.r-project.org). Somatic mutations and somatic copy number alternations (CNAs) data were downloaded from the TCGA database. Copy number alternations associated with risk scores were analyzed using GISTIC 2.0 (https://gatkforums.broadinstitute.org). Adjusted P values were obtained by False Discovery Rate (FDR) correction. P values and adjusted P values of less than 0.05 were considered statistically significant.

## Results

### Data preprocessing and DEGs screening

**Figure 1:**
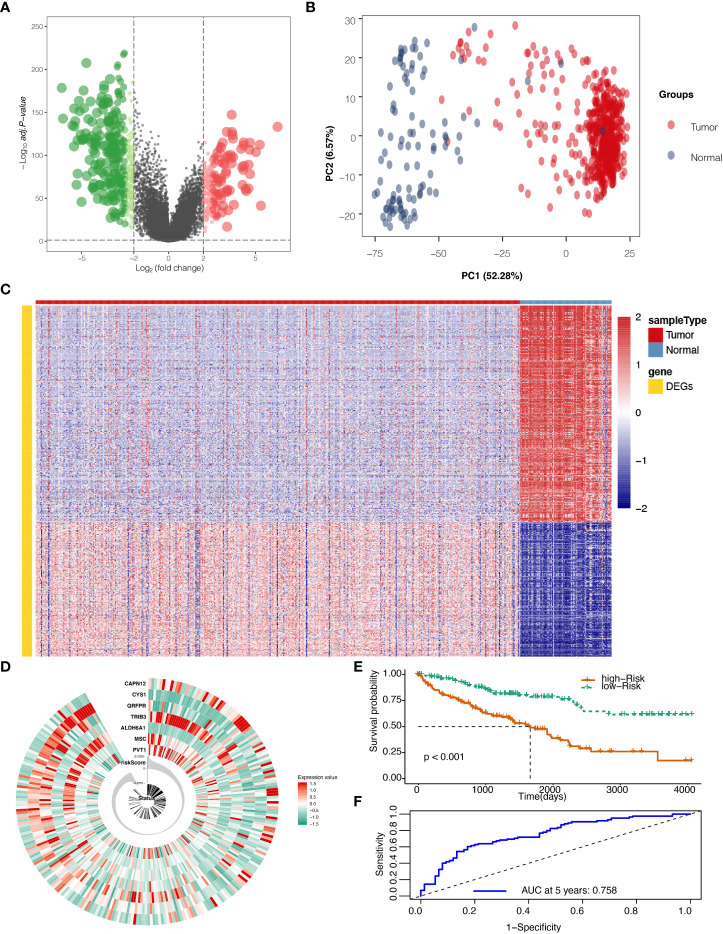
Differentially expressed genes (DEGs) screening and localization. **(A)** Volcano plot for DEGs with adjusted P values (FDR correction) less than 0.05. **(B)** Principal component analysis (PCA) to validate screening results. **(C)** Heat map result for DEGs screening. **(D)** Risk scores in the Cancer Genome Atlas (TCGA) train set, patient survival, and expression of 7 DEGs in the train set. **(E)** Risk score and patients’ survival probabilities in the TCGA train set. **(F)** Receiver operating characteristic curve (ROC) of the risk score in the TCGA train set.

The flow chart of this study is shown in **Supplementary Figure S1A**. After mining the data in the GTEx and TCGA databases, 528 KIRC and 100 normal samples were gathered and clustered to screen for differentially expressed genes between cancer and normal tissue. With a threshold of logFC>2 and adjust P ≦0.05, 594 genes (**Table S1**) were found to be differentially expressed, among which 227 genes were up-regulated, and 367 genes were down-regulated (**Figure 1A**). Those DEGs in KIRC and normal tissue can be separated by PCA (**Figure 1B**). The heatmap shows that the DEGs effectively separate KIRC and normal tissue (**Figure 1C**).

### Development of the risk score with TCGA train set

To calculate the risk score, five hundred twenty-eight samples from TCGA were randomly separated into 264 and 264. In the train set containing 264 patients, lasso regression was adopted to analyze the data. After multiplying gene expression with LASSO coefficients, we came to seven prognostic genes: PVT1, MSC, ALDH6A1, TRIB3, QRFPR, CYS1, and CAPN12 (**Table S2**). The risk score was then calculated for patients using seven prognostic genes between high and low-risk groups set at the median value (**Figure 1D**). Risk score=0.0009* PVT1 (gene expression level) + 0.0015*MSC + -0.0029*ALDH6A1 + 0.0022*TRIB3 + -0.0003*QRFPR +-0.0038*CYS1 + 0.0011* CAPN12. The calculated risk score ranged from -0.875 to 0.733 and had a median value of -0.007, in which the patients were grouped into a high-risk group and a low-risk group based on the median value of the risk score. In the train set, the high-risk and low-risk groups presented significantly different survival probabilities (**Figure 1E**) with an AUC of 0.758 in the time-dependent ROC curve at five years (**Figure 1F**).

### Validation of the risk score with TCGA and GEO data

**Figure 2:**
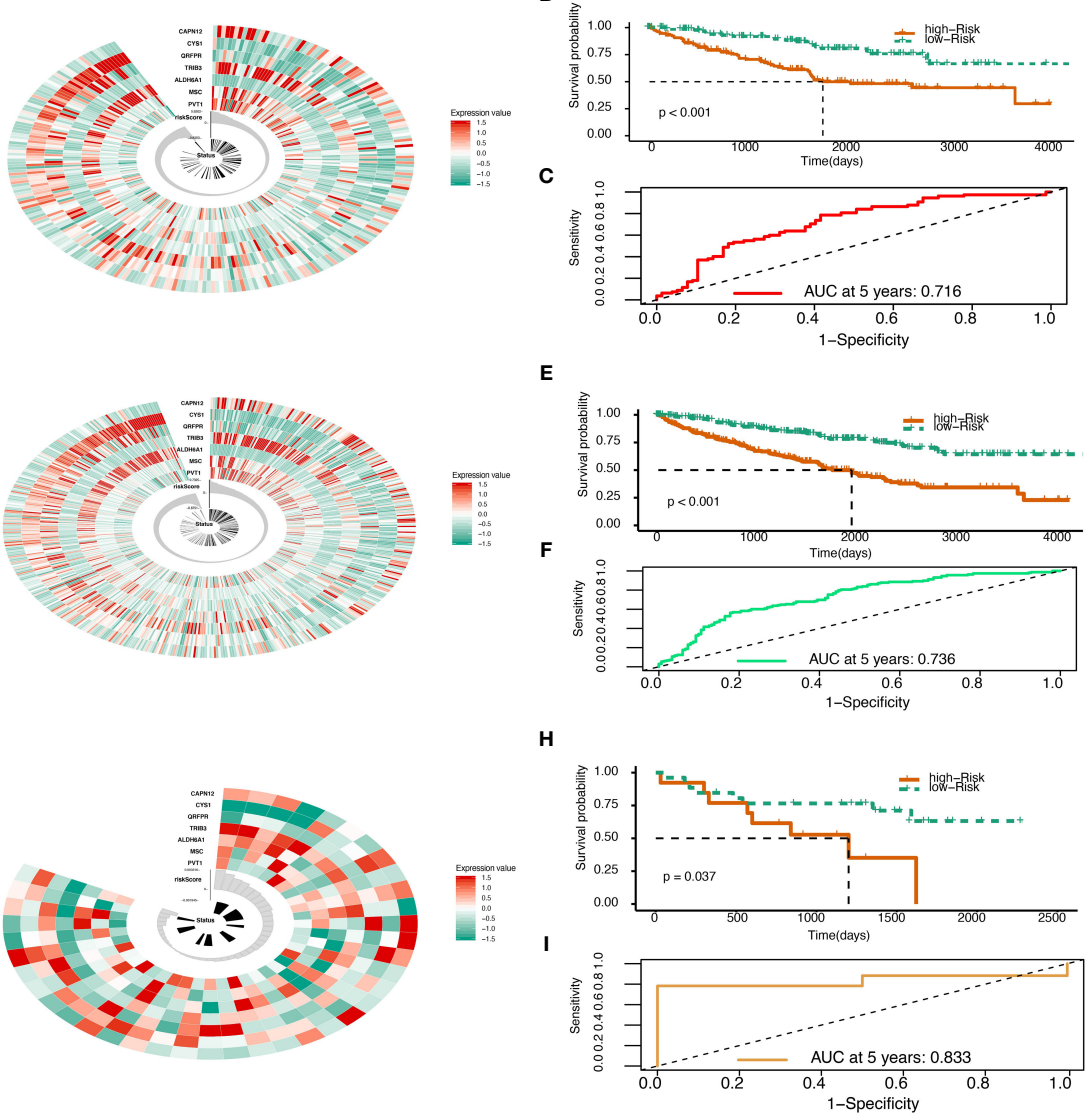
Risk score validation. **(A)** Risk scores in the TCGA test set, patient survival, and expression of 7 DEGs in the test set. **(B)** Risk score and patients’ survival probabilities in the TCGA test set. **(C)** The ROC of risk scores in the TCGA test set. **(D)** Risk scores in the TCGA sum set, patient survival, and expression of 7 DEGs in the sum set. **(E)** Risk score and patients’ survival probabilities in the TCGA sum set. **(F)** The ROC of the risk scores in the TCGA sum set. **(G)** Risk scores in the Gene Expression Omnibus (GEO) validation set, patient survival, and expression of 7 DEGs in the validation set. **(H)** Risk scores and patients’ survival probabilities in the GEO validation set. **(I)** The ROC of risk scores in the GEO set.

The Risk Score was calculated in the test set (**Figure 2A**). With the cutoff of risk score, survival probability between the high and low-risk score groups is statistically significant (P<0.001) with an AUC value of 0.716 (**Figures 2B, C**). When summed up, the risk score was further calculated with a P value of less than 0.001 between high and low-risk score groups and an AUC of 0.833 (**Figures 2D–F**). The model was then tested using GEO data in microarray GSE29609 from platform GPL1708. The difference between high and low-risk score groups in GEO data analysis was also statistically significant (P=0.037) with an AUC of 0.833 (**Figures 2G–I**). All model evaluation was based on time-dependent ROC at five years.

### Genomic alterations and gene set enrichment analyses

**Figure 3:**
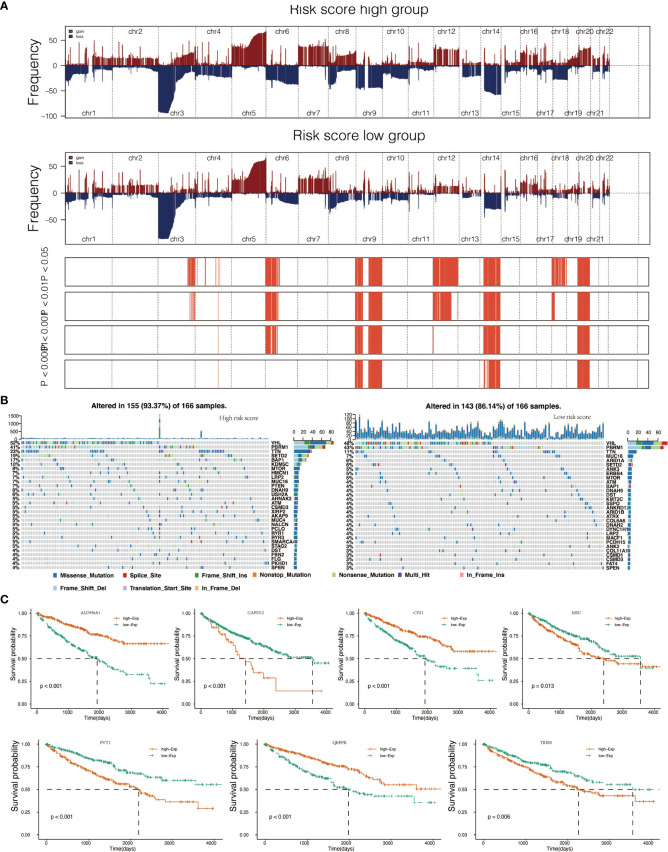
Genomic alterations in score low vs. high clusters and DEGs expression in cell lines. **(A)** Copy number variation (CNV) profile in the low score group and CNV profile in the high score group. **(B)** Genomic event enrichment in the low score cluster and genomic event enrichment in the high score cluster. **(C)** Kaplan-Meier overall survival (OS) of patients and expression level of seven prognostic genes.

To determine whether risk score levels were associated with specific genomic characteristics, we performed CNV and somatic mutation analysis using the TCGA dataset (**Table S13**). In high-score samples, frequently amplified genomic regions included oncogenic driver genes such as RSRC1 (3q25.32, p<0.001), SLC2A9 (4p16.1, p<0.001), EXOC2 (6p25.3, p<0.001), EGFR (7p11.2, p<0.001), and ERC1 (12p13.33, p<0.001) (**Figure 3A**). In contrast, deleted regions contained tumor suppressor genes including PTENP1 (9p13.3, p<0.001), FAM138C (9p24.3, p<0.001), and OR4K15 (14q11.2, p<0.001) (**Figure 3A**). In low-score samples, most amplified and deleted genomic regions were similar to those in high-score models. Analysis of somatic mutation profiles based on risk score levels revealed a high frequency of mutations in SETD2 (19%, p < 0.001), BAP1 (17%, p < 0.001), and KDM5C (10%, p < 0.01) in the high-score group (n = 166) (**Figure 3B**; **Table S14**). Genomic event enrichments were identified in either the low-score or high-score groups, respectively (**Figure 3B**).

### Consensus clustering of seven prognostic genes

**Figure 4:**
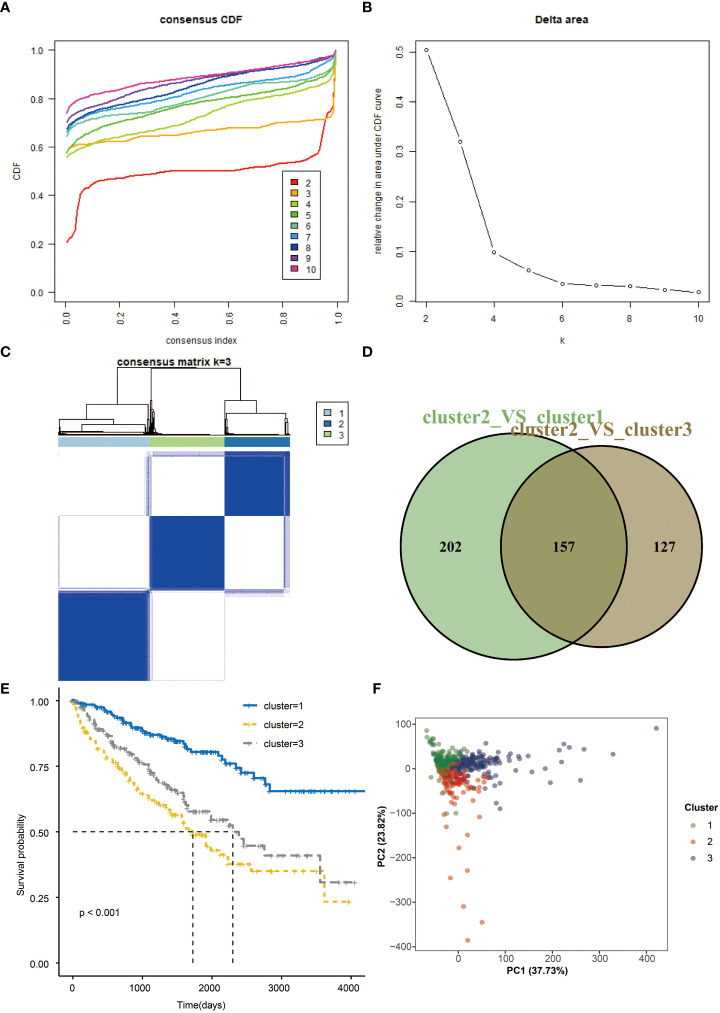
Consensus clustering and overall survival in three subgroups. **(A)** Consensus clustering cumulative distribution function (CDF) for k=2 to 10 in TCGA data. **(B)** Relative change in area under CDF curve for k=2 to 10. **(C)** Consensus matrixes of TCGA for each k=3. **(D)** Venn plots the two DEGs groups **(E)** Kaplan-Meier overall survival (OS) curves using TCGA data. **(F)** PCA results for two groups of patients.

Consensus clustering of the seven prognostic genes identified three clusters of KIRCs in the TCGA dataset with distinct clinical outcomes, clinical features, and pathological features (**Figures 4A, B**). In the TCGA dataset, according to the expression similarity, k=3 was selected with clustering stability rising from k=2 to 10 in the TCGA dataset since the consensus cumulative distribution function (CDF) curve was flattest at k=3. Thus, consensus and cluster confidence are also maximal at this k (**Figure 4C**). The Venn diagram further showed the DEGs among three clusters (**Figure 4D**). Among the three groups, survival probability is distinctively separated (**Figure 4E**), which was also confirmed by PCA (**Figure 4F**).

### Gene set variation analysis and geneset enrichment analysis

**Figure 5:**
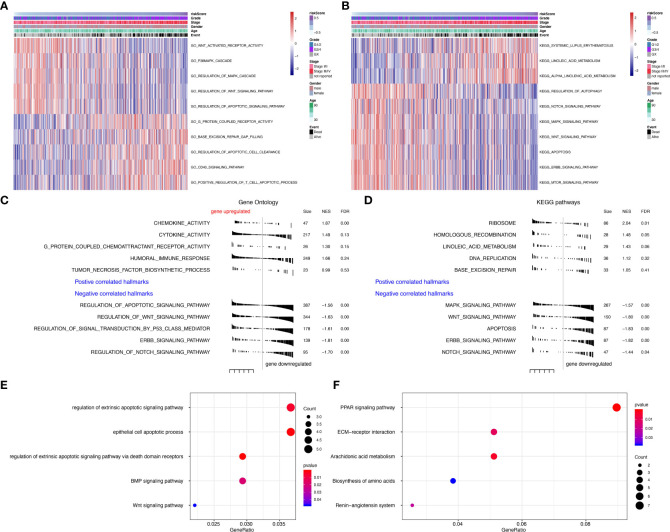
Gene set variation analysis (GSVA) and Geneset enrichment analysis (GSEA) in the TCGA dataset. **(A)** Gene Ontology (GO) results based on GSVA in TCGA dataset. **(B)** Kyoto Encyclopedia of Genes and Genomes (KEGG) results based on GSVA in TCGA dataset. **(C)** GO results based on GSEA in TCGA dataset. **(D)** KEGG results based on GSEA in TCGA dataset. **(E)** GO enrichment analyses in TCGA dataset. **(F)** KEGG enrichment analyses in TCGA dataset.

To further explore the function of seven prognostic genes, GSVA was conducted using TCGA data (**Tables S3, S4**). The most enriched GO functions are the regulation of the Wnt signaling pathway, regulation of MAPK cascade, regulation of apoptotic signaling pathway, base excision repair gap filling, positive regulation of T cell apoptotic process, etc. (**Figure 5A**). Analyses in KEGG pathways revealed that systemic lupus erythematosus, linoleic acid metabolism, regulation of autophagy, Notch signaling pathway, MAPK signaling pathway, Wnt signaling pathway, apoptosis, ERBB signaling pathway, and mTOR signaling pathway were correlated with the seven prognostic genes (**Figure 5B**). GSEA (**Tables S5, S6**) further confirmed that the seven predictive genes were enriched in GO pathways such as cytokine activity, humoral immune response, regulation of apoptotic signaling pathway, regulation of Wnt signaling pathway, regulation of signal transduction by p53 class mediator, ERBB signaling pathway, and regulation of Notch signaling pathway (**Figure 5C**). As for KEGG pathways, seven prognostic genes were enriched in the ribosome, MAPK signaling pathway, Wnt signaling pathway, apoptosis, ERBB signaling pathway, and Notch signaling pathway (**Figure 5D**). The dot plot of GO and KEGG enrichment analysis (**Tables S7, S8**) further revealed that high risk scores were associated with regulation of extrinsic apoptotic signaling pathway, epithelial cell apoptotic process, BMP signaling pathway, and Wnt signaling pathway in GO pathways (**Figure 5E**), while the risk score was enriched in PRAR signaling pathway, ECM-receptor interaction, arachidonic acid metabolism, biosynthesis of amino acids and the renin-angiotensin system in KEGG pathways (**Figure 5F**). The correlation between seven prognostic genes and GO pathways was shown in **Supplementary Figure S2B**, while the correlation between seven predictive genes and KEGG pathways was shown in **Supplementary Figure S2C**.

### Immunological function analyses

**Figure 6:**
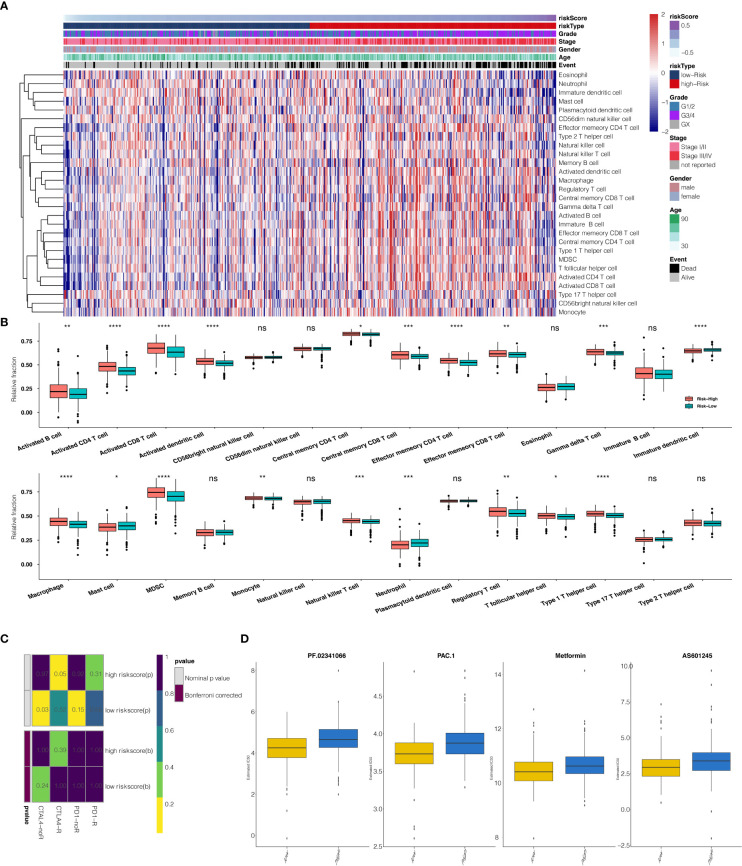
**(A)** The heatmap illustrates the association between risk scores and immune infiltrating cells. **(B)** Correlation between risk score and immune infiltrating cells. **(C)** Submap analysis showed that a high-risk score could be more sensitive to the CTLA-4 inhibitor (Nominal p-value = .05 *P<0.05; **P<0.01; ***P<0.001; ****P<0.0001, ns, not statistically significant). **(D)** The box plots show the estimated IC50 for PF.02341066, PAC.1, Metformin, and AS601245 for high-risk scores and low-risk scores.

The risk scores calculated from prognostic genes are correlated with immune infiltrating cells in the tumor microenvironment (TME). High-risk scores were significantly associated with the relative expression levels of macrophage, MDSC, activated CD4 T cell, activated CD8 T cell, and type 1 T helper cell. In contrast, low-risk scores were correlated with the relative expression levels of immature dendritic cells and neutrophils (**Figures 6A, B**, correlation > 0.2, P<0.001). Three clusters identified by the seven prognostic genes were also significantly correlated with regulating immune cells in TME (**Figure S4A**). The correlation between seven predictive genes and immune infiltrating cells was shown in **Supplementary Figure S2A**, in which seven genes are highly correlated with multiple immune infiltrating cells.

### Survival impact of prognostic genes

When comparing survival probabilities between patients with different expression levels of the seven prognostic genes, we found that high ALDH6A1, CYS1, and QRFPR were associated with worse overall survival (OS). In contrast, increased expression of CAPN12, PVT1, MSC, and TRIB3 indicated a better prognosis (**Figure 3C**). The time-dependent ROC curve at five years of these seven prognostic genes was shown in **Figure S1B**. We next conducted the survival analysis of the risk score. High-risk scores were associated with worse OS in different age groups, sex, grade, and stage (**Figure S3A**). The expression pattern of risk scores in various prognostic factors was shown in **Figure 6B**, in which high-risk scores were significantly correlated with older patients, male patients, KIRC at grade 4, and KIRC at stage iv. We also revealed that the high-risk scores connected with T4N1M1 KIRC based on the TNM location (**Figure S3B**). High-risk scores were also related to worse disease-specific survival (DSS) and progressive-free survival (PFS) in the KIRC cohort (**Figure S4B**). We next verified the seven prognostic genes in kidney renal papillary cell carcinoma (KIRP), in which high-risk scores indicated worse OS, DSS, and PFS in the KIRP cohort (**Figure S4C**).

### Prediction of risk scores for immunotherapy and chemotherapy

The potential response to immunotherapy in TCGA based on the TIDE algorithm was evaluated, in which our results showed that patients with high-risk scores had a better answer to anti-Cytotoxic T-Lymphocyte Associated Protein (CTLA4) immunotherapy than those with low-risk scores (Nominal p-value = .05) (**Figure 6C**). Considering that chemotherapy is the standard way to treat ccRCC, we tried to assess the response of patients with different risk scores to various chemo drugs. We could observe a significant difference in the estimated IC50 between high-risk scores and low-risk scores for PF.02341066, PAC.1, Metformin, and AS601245, which low-risk scores could be more sensitive to commonly administered chemotherapies (P <.001 for PF.02341066, PAC.1, Metformin, and AS601245, respectively) (**Figure 6D**).

### Development of the prognostic model with TCGA data

**Figure 7:**
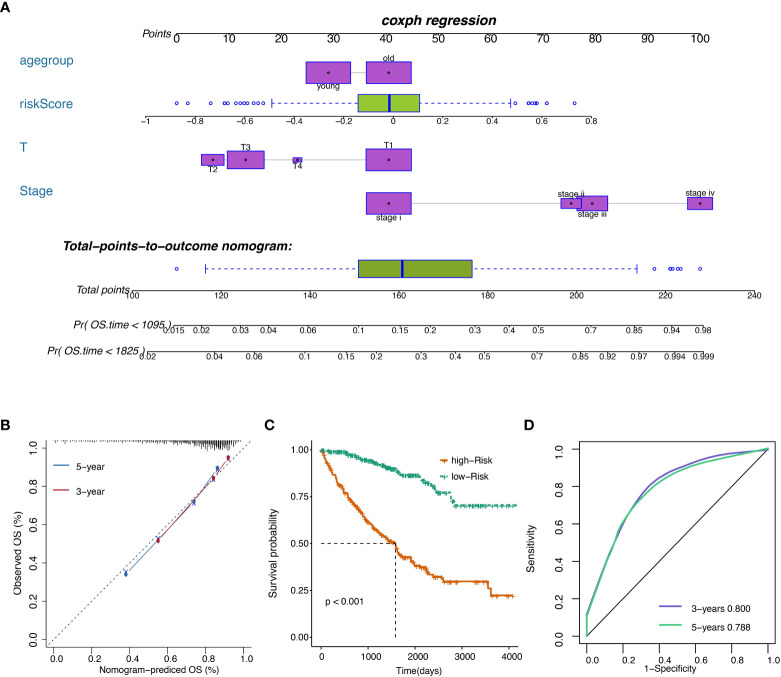
**(A)** Nomogram of the prognostic model. **(B)** Model evaluation results. **(C)** OS of patients with high or low overall risks. **(D)** ROC of the model in TCGA datasets.

The risk score was subsequently validated as an independent prognostic marker after adjusting for several risk factors, including age group, primary tumor lesion, and stage in univariate and multivariate Cox regression analysis concerning OS, DSS, and PFS (**Tables S8, S10, S11**. respectively) in the TCGA dataset. The predictive model we built includes risk score, age group, primary tumor lesion, and stage (**Figure 7A**). At both the three-year and five-year survival, the model had satisfying results in the evaluation nomogram (**Figure 7B**). Survival difference between high and low-risk patients was statistically significant (**Figure 7C**). In TCGA data, the AUC at three years is 0.800 and the AUC at five years is 0.788 in the sensitivity test (**Figure 7D**).

### CAPN12 and MSC suppress cell proliferation in ccRCC cells

**Figure 8:**
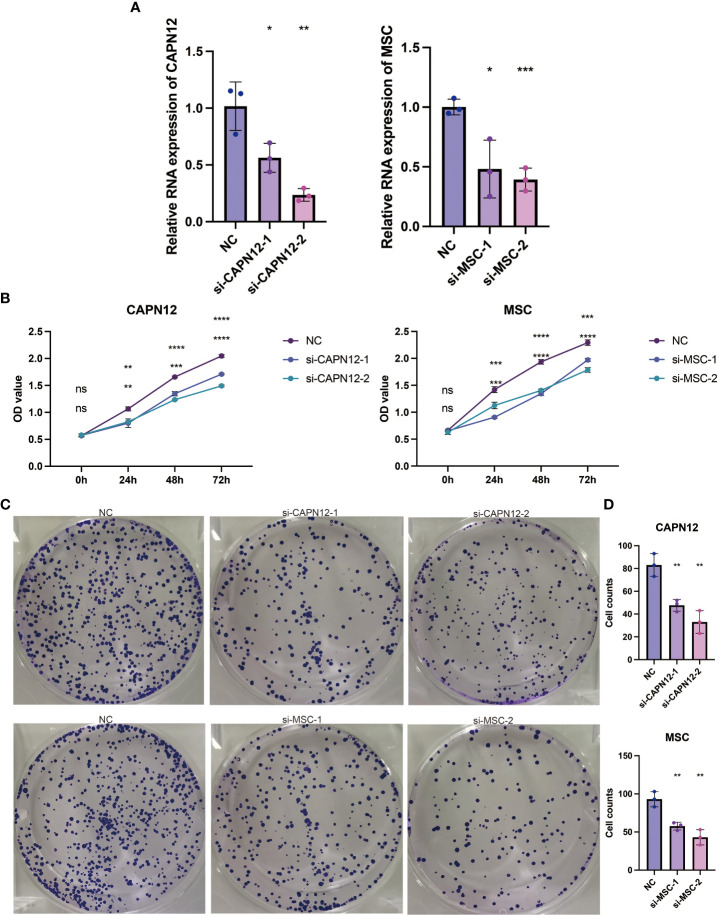
**(A)** qRT-PCR assays for the CAPN12 and MSC levels in 786-O cells transfected with two different siRNAs targeting CAPN12 and MSC (si#1 and is #2), respectively. Tukey HSD test. *P <.05, **P <.01, ***P <.001, ****P <.0001, ns, not statistically significant. **(B)** CAPN12 and MSC knockdown cell proliferation were measured using CCK-8 assay. **(C)** Plate clonality assays measuring the impact on cell clonality and cell cycle in 786-O cell line after silencing CAPN12 and MSC. **(D)** Statistical analysis in plate clonality assay.

According to the endogenous CAPN12 and MSC expression level, two independent siRNAs targeting CAPN12 and MSC were transfected into the 786-O cell line with relatively high expression of CAPN12 and MSC. The efficiency of the knockdown of CAPN12 and MSC expression was validated by qRT-PCR (**Figure 8A**, p<0.001). It was demonstrated that the proliferative capacity of 786-O cells was significantly repressed by CAPN12 and MSC knockdown (**Figure 8B**). Plate clonality assays revealed the remarkable suppression of cell clonality and cell cycle in the 786-O cell line after silencing CAPN12 and MSC (**Figures 8C, D**).

### Pan-cancer analysis on CAPN12 and MSC

**Figure 9:**
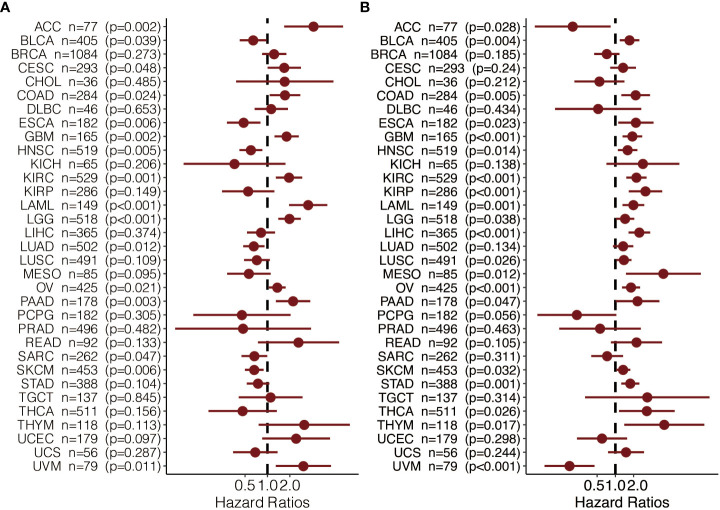
The prognostic value of **(A)** CAPN12 and **(B)** MSC in pan-cancer.

**Figure 10:**
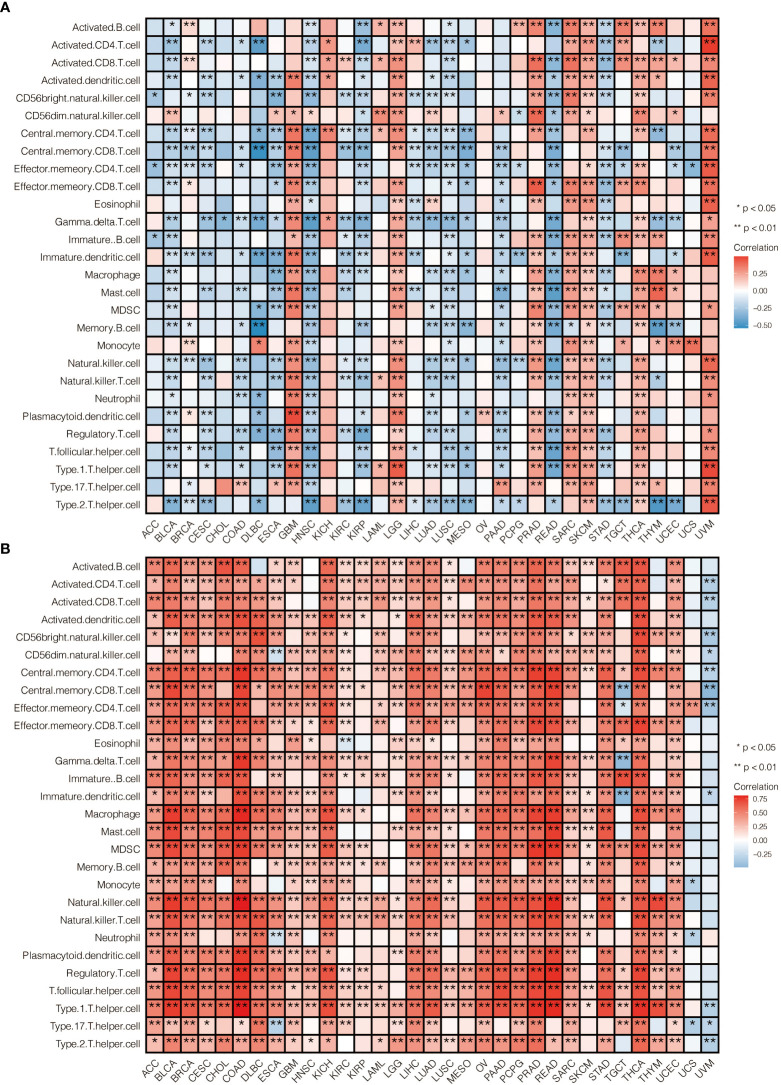
The immune infiltration pattern of **(A)** CAPN12 and **(B)** MSC in pan-cancer.

To further explore the prognostic value and immune infiltration pattern of CAPN12 and MSC, pan-cancer samples from TCGA were used for analysis. CAPN12 (**Figure 9A**) and MSC (**Figure 9B**) were hazardous markers in most cancer types. Besides, CAPN12 (**Figure 10A**) and MSC (**Figure 10B**) correlated with the infiltration of multiple immune cells in most cancer types. These results suggested that CAPN12 and MSC could be predictive markers of prognosis and immune infiltration in cancer.

## Discussion

High mortality and recurrence rates have made ccRCC the most devastating tumor in the urinary system. Previous tic stratification and treatment strategies studies have focused on investigating single potential prognostic biomarkers for ccRCC (17–19). However, none has been immensely satisfying. As high-throughput sequencing and bioinformatics quickly develop, mining the large volume of genetic data has been increasingly appealing to researchers. After data mining, a prognostic model built on genetic profiles of ccRCCs poses significance in developing a prong.

In this study, specifically, after comparing global gene expression in ccRCC samples and controls, 594 DEGs were identified. After univariate and lasso regression analyses, 7 out of 594 DEGs were considered prognostic value: PVT1, MSC, ALDH6A1, TRIB3, QRFPR, CYS1, and CAPN12. Notably, high ALDH6A1, CYS1, and QRFPR were associated with worse OS, while high expressions of CAPN12, PVT1, MSC, and TRIB3 showed statistically significant survival benefits.

Calpains (CAPNs), a family of cysteine proteases, have been demonstrated to play a critical role in cancer development and progression and the insufficient response to cancer therapiesStarsky (20). CAPN12, a gene involved in apoptosis and suppressed by p53, is the critical determinant of anti-tumor response in medulloblastoma (21). Long non-coding RNA plasmacytoma variant translocation 1 (PVT1), up-regulated in various human cancers, inhibits renal cancer cell apoptosis *via* up-regulating Mcl-1 (22) and downregulating miR-16-5p (23). The knockdown of PVT1 induces apoptosis and cell cycle arrest through the epidermal growth factor receptor pathway (24). Multiple studies have also proved that PVT1 predicts unfavorable prognosis in patients with ccRCC (25, 26). MSC, also belonging to the lncRNA family, activates the Wnt/β-catenin signaling pathway to modulate cell proliferation and migration in ccRCC *via* miR-3924/WNT5A (27).

ALDH6A1, regulated by transcription factor HNF4A, has already been verified in other bioinformatics analyses to suppress tumorigenic capability in ccRCC and to be a prognostic biomarker (28, 29).

Tribbles pseudokinase 3 (TRIB3), a member of the mammalian pseudokinase tribbles family, is involved in multiple biological processes, including tumor progression. The previous study has revealed that TRIB3 promoted the proliferation and invasion of ccRCC *via* activating MAPK signaling pathway (30).

QRFPR, also named GPR103, activates glutamine RF−amide peptide (QRFP), is over-expressed in human prostate cancer, and stimulates the neuroendocrine differentiation and the migration of androgen-independent prostate cancer cells (31, 32).

CYS1 mutation on chromosome 2p25 has been proven to be a candidate for recessive cystic kidney disease (33). CAPN12 and MSC were selected for *in vitro* gene silencing among the seven prognostic genes. The cell proliferation assay demonstrated that the proliferative capacity of 786-O cells was significantly repressed by CAPN12 and MSC knockdown, revealing the tumorigenic role of CAPN12 and MSC.

Further geneset variation analysis was conducted in these seven prognostic genes to explore involved signaling pathways. GO analysis revealed that predictive genes are primarily enriched in the Wnt signaling pathway, MAPK cascade, regulation of apoptotic signaling pathway, base excision repair gap filling, positive regulation of T cell apoptotic process, etc. KEGG pathway revealed systemic lupus erythematosus, linoleic acid metabolism, regulation of autophagy, Notch signaling pathway, MAPK signaling pathway, Wnt signaling pathway, apoptosis, ERBB signaling pathway, and mTOR signaling pathway were correlated with the seven prognostic genes. GSEA further confirmed that these seven predictive genes were involved in the tumor-genic process. All these results support the significance of predictive genes in ccRCC.

Hence, risk scores were calculated for each patient based on the seven prognostic genes. When The high-risk group showed a significant survival disadvantage when we separated patients according to the median risk scores contrast, patients with low-risk scores had better responses to chemotherapy. The risk score was further validated in the TCGA test set, TCGA sum set, and GEO data set. High-risk patients showed significantly worse survival in all data sets than low-risk patients. Before we developed a prognostic model, consensus clustering was adopted to evaluate the predictive genes, and the clustering findings suggested that predictive genes are closely related to survival probability. Patients with high-risk scores also had infiltrating immune cell levels similar to those in cluster 2. Given that the increased risk score group and cluster 2 predicted worse survival, the validity of these genes was supported from another aspect.

Moreover, the risk score was correlated with immune cell expression. High-risk scores were significantly associated with the macrophage, MDSC, activated CD4 T cell, activated CD8 T cell, and type 1 T helper cell expression.

In contrast, low-risk scores were associated with immature dendritic cells and neutrophils, which implicates a suppression in both the innate and acquired immune response system. This finding would open a gate to targeting the immune system to fight ccRCC. Though the activated CD4 T cell and activated CD8 T cell expression increased under such a situation, it could represent positive feedback from a tumor attack.

Interestingly, when examining the genomic alteration profiles of low- and high-risk groups, we found that VHL expression was much higher in the low-risk group. Since VHL plays a tumor-suppressing role, this connection validates the value of the calculated risk score from another perspective. However, further studies are in need to explore the causality in between.

Next, a prognostic model containing risk score, age group, primary tumor lesion, and stage was developed satisfyingly. The remarkable ROC value indicates that the predictive model could be an essential predicting tool. Although ccRCC patient overall survival is influenced by age group, primary tumor lesion, and stage, our risk score adds value to disease prognosis independently by categorizing patients into groups with distinct survival probability. Notably, the high-risk score was significantly correlated with male patients, KIRC at grade 4, KIRC at stage iv, and T4N1M1 based on the TNM location.

## Conclusion

Some prognostic models(Wang et al., 2019; Zhang et al., 2019) focus on various ccRCC prognostic factors, such as DNA methylation-driven genes and metastasis-associated predictive genes. However, only some models are acknowledged as a golden standard due to the complex nature of ccRCC, which leaves much space for further research. Seven prognostic genes were eliminated from this study’s analysis of data from two databases (TCGA and GEO), all of which were most likely to be highly linked with the onset and progression of ccRCC. We subsequently conducted extensive investigations to create a full prognostic model for ccRCC patients, offering a reliable signature for prognosis prediction and supporting data for drug discovery against these predictive genes. Although knockdown cell RNA-seq was not performed to examine expression profiles and the knockout specificity, qRT-PCR was used to confirm the knockout of CAPN12 and MSC. Additionally, further investigation based on a large cohort is required to fully understand the exciting findings that T2, T3, and T4 were linked to better outcomes (HR 1). Due to this limitation, there is still space for additional validation.

## Data availability statement

The original contributions presented in the study are included in the article/**Supplementary Material**. Further inquiries can be directed to the corresponding author.

## Author contributions

GX conducted the data analysis. SW provided funding support, and XC supervised and revised the study. All authors contributed to the article and approved the submitted version.

## Supplementary material

Supplementary Table 1 The list of differentially expressed genes (DEGs).

Supplementary Table 2 LASSO results of prognostic genes.The list of differentially expressed genes (DEGs).

Supplementary Table 3 Gene set variation analysis (GSVA) of the risk score in GO.

Supplementary Table 4 Gene set variation analysis (GSVA) of the risk score in KEGG.

Supplementary Table 5 Gene set enrichment analysis (GSEA) of the risk score in GO

Supplementary Table 6 Gene set enrichment analysis (GSEA) of the risk score in KEGG

Supplementary Table 7 GO enrichment analysis of risk score.

Supplementary Table 8 KEGG enrichment analysis of risk score

Supplementary Table 9 Univariate and multivariate analysis regarding OS in the prognostic model.

Supplementary Table 10 Univariate and multivariate analysis regarding DSS in the prognostic model.

Supplementary Table 11 Univariate and multivariate analysis regarding PFS in the prognostic model.

Supplementary Table 12 The relative abundance of 28 immune cell types in each sample.

Supplementary Table 13 Significantly different CNV regions of risk score.

Supplementary Table 14 Significantly different mutated genes of high and low-risk score groups.
