# Supplementary material for: An immune infiltration-related prognostic model of kidney renal clear cell carcinoma with two valuable markers: CAPN12 and MSC
Source: Front Oncol. 2023 Mar 21;13:1161666. doi: 10.3389/fonc.2023.1161666 (PMC10071012; doi:10.3389/fonc.2023.1161666)
Supplement: Supplementary file 1 [file DataSheet_1.docx]

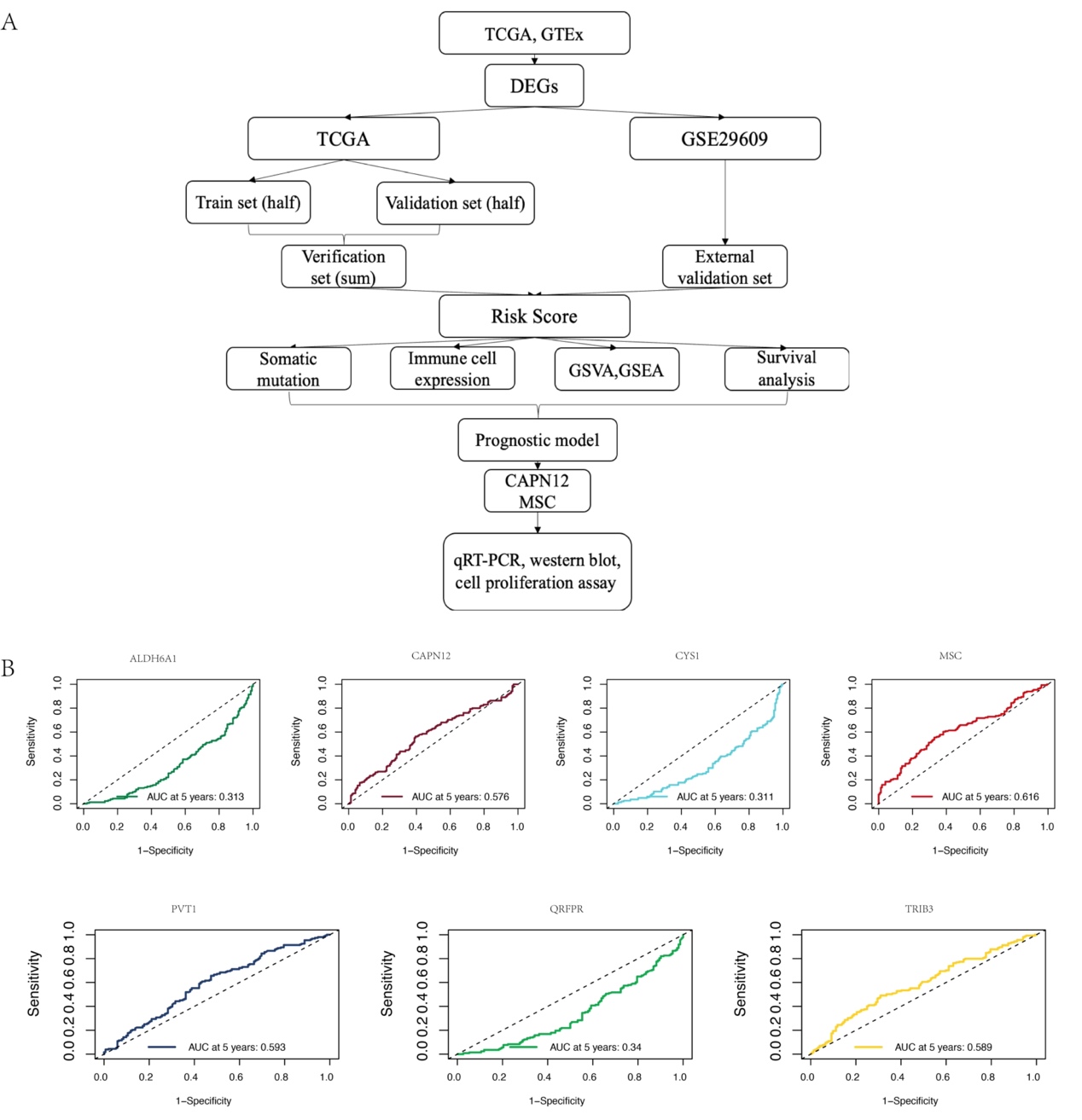


Figure S1. Relationship between DEGs and OS. A. Flow chart of the study. B. ROC of seven prognostic genes assessing 5-year survival.


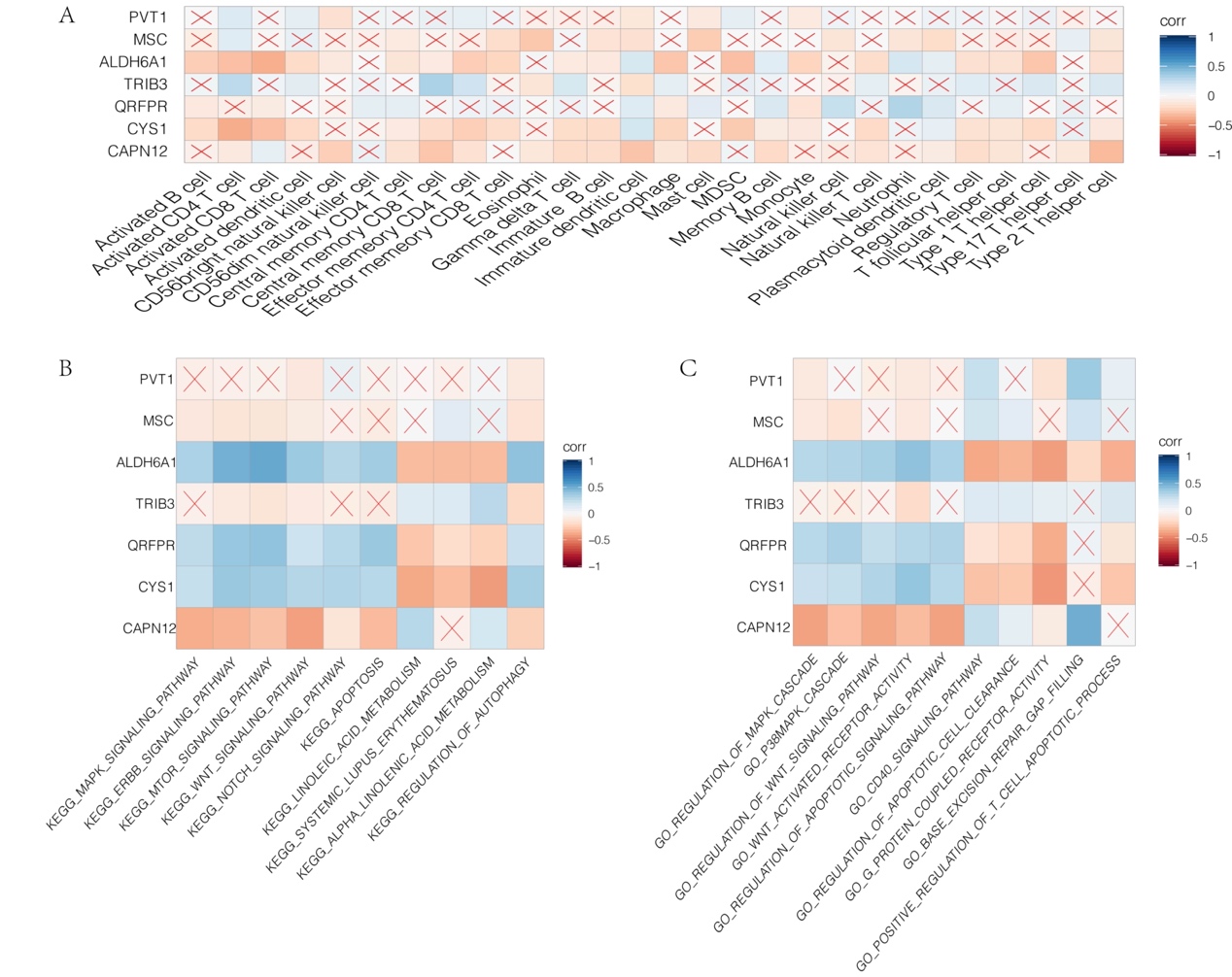


Figure S2. A. Correlation between seven prognostic genes and immune infiltrating cells. B. Correlation between seven prognostic genes and GO pathways. C. Correlation between seven prognostic genes and KEGG pathways. Correlation analysis was based on Spearman correlation.


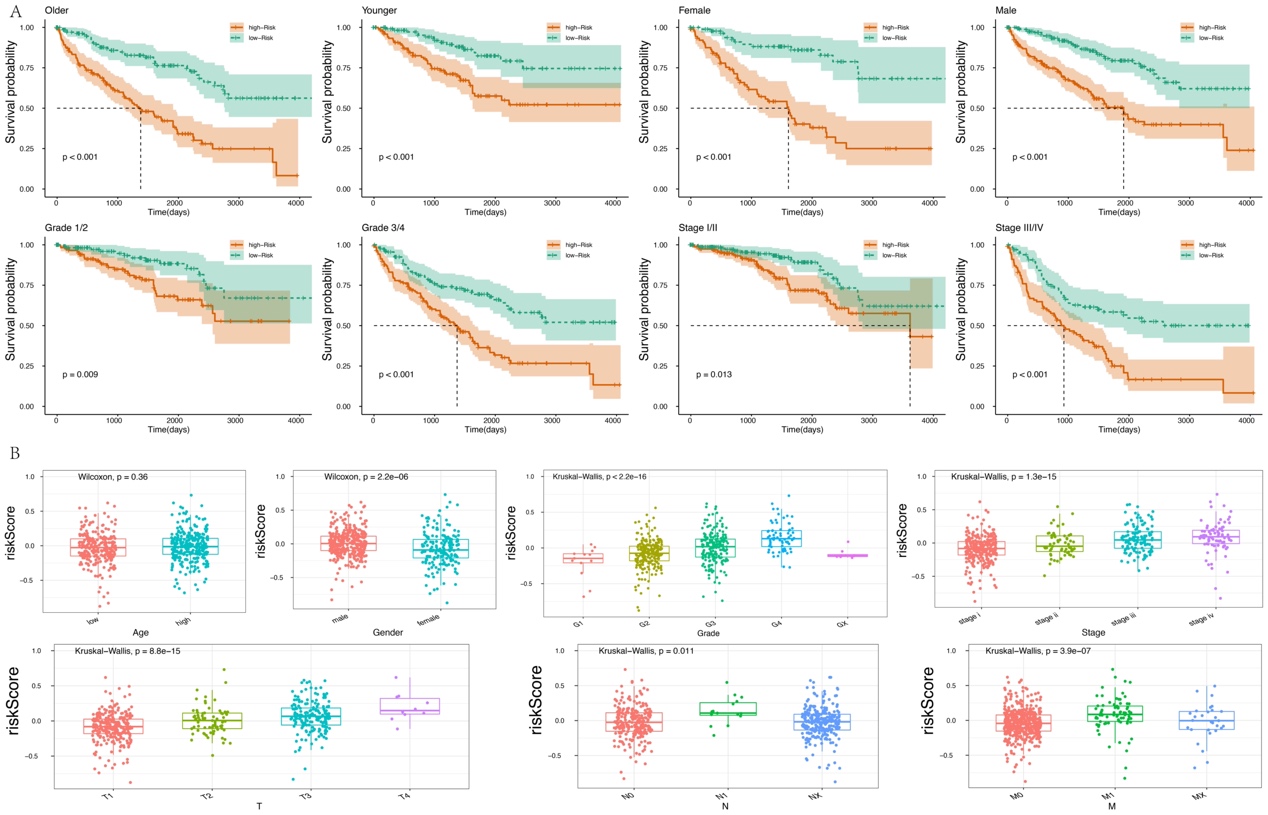


Figure S3. Relationship between risk score, age, gender, tumor grade, and tumor stage. A. Kaplan-Meier overall survival (OS) of patients and risk score regarding age, gender, tumor grade, and tumor stage. B. The differences in the risk score in age, gender, tumor grade, tumor stage, and TNM stages.


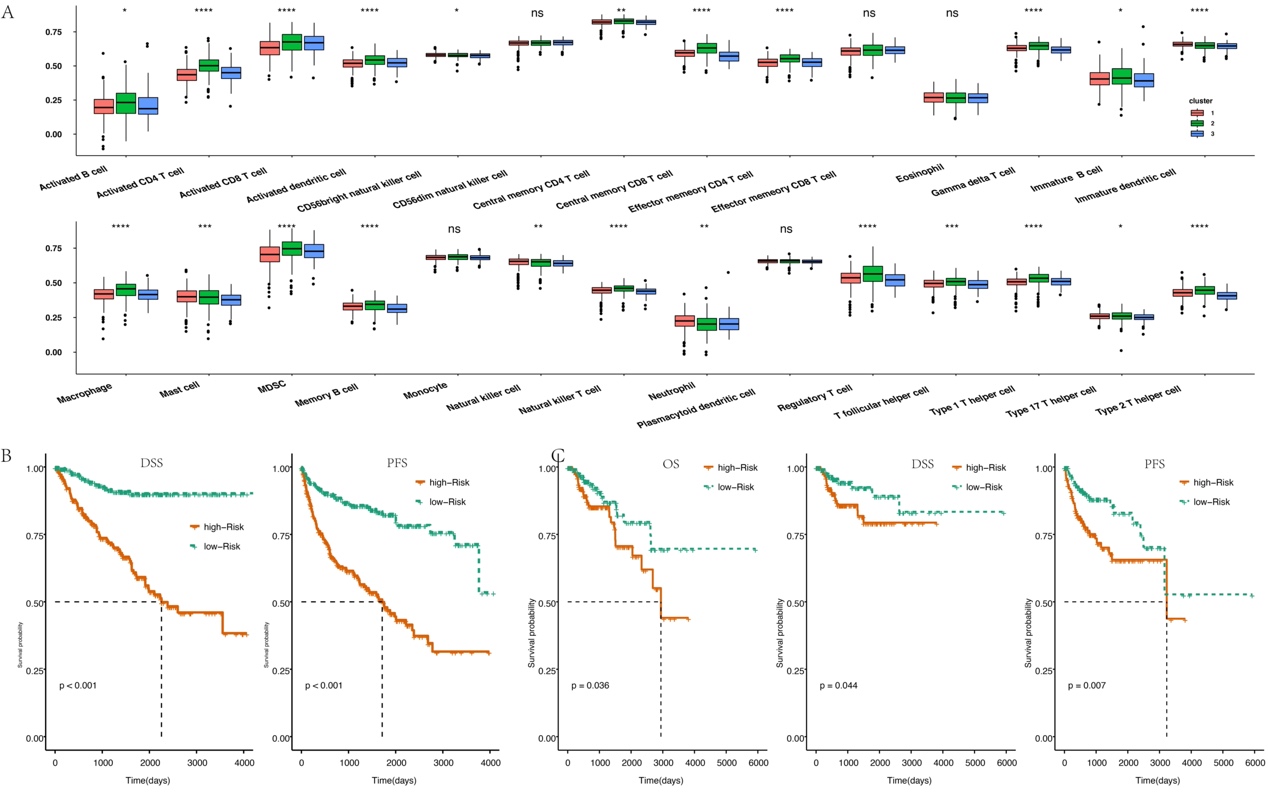


Figure S4. A. The expression difference of immune infiltrating cells in three identified clusters. B. Relationship between risk score, disease-specific survival (DSS), and progression-free survival (PFS) in the KIRC cohort. C. Relationship between risk score, OS, DSS, and PFS in KIRP cohort.
