# Supplementary material for: An immune infiltration-related prognostic model of kidney renal clear cell carcinoma with two valuable markers: CAPN12 and MSC
Source: Front Oncol. 2023 Mar 21;13:1161666. doi: 10.3389/fonc.2023.1161666 (PMC10071012; doi:10.3389/fonc.2023.1161666)
Supplement: Supplementary Table 2 — LASSO results of prognostic genes.The list of differentially expressed genes (DEGs). [file Table_2.docx]

| Final Gene | Active. Coefficients |
| --- | --- |
| PVT1 | 0.000929 |
| MSC | 0.001517 |
| ALDH6A1 | -0.002874 |
| TRIB3 | 0.0021973 |
| QRFPR | -0.000255 |
| CYS1 | -0.003767 |
| CAPN12 | 0.0010700 |
