# Supplementary material for: An immune infiltration-related prognostic model of kidney renal clear cell carcinoma with two valuable markers: CAPN12 and MSC
Source: Front Oncol. 2023 Mar 21;13:1161666. doi: 10.3389/fonc.2023.1161666 (PMC10071012; doi:10.3389/fonc.2023.1161666)
Supplement: Supplementary Table 9 — Univariate and multivariate analysis regarding OS in the prognostic model. [file Table_9.docx]

| Characteristic | P | HR | 95%CI | P | HR | | 95%CI |
| --- | --- | --- | --- | --- | --- | --- | --- |
| Univariate Multivariate | | | | | | | |
| Riskscore | < 0.001 | 15.97 | 9.31-30.68 | < 0.001 | 9.10 | 4.30-19.28 | |
| Agegroup (Young) | < 0.001 | 0.56 | 0.41-0.76 | 0.002 | 0.61 | 0.45-0.84 | |
| Gender (Male) | 0.804 | 0.96 | 0.70-1.31 | 0.415 | 0.87 | 0.62-1.22 | |
| Tumor |  |  |  |  |  |  | |
| T2 | 0.101 | 1.53 | 0.92-2.55 | 0.008 | 0.20 | 0.06-0.66 | |
| T3 | < 0.001 | 3.12 | 2.21-4.41 | 0.022 | 0.31 | 0.12-0.85 | |
| T4 | < 0.001 | 10.82 | 5.30-22.09 | 0.057 | 0.31 | 0.09-1.04 | |
| Metastasis |  |  |  |  |  |  | |
| M1 | < 0.001 | 4.22 | 3.08-5.79 | 0.247 | 0.46 | 0.12-1.71 | |
| MX | 0.991 | 0.99 | 0.31-3.14 | 0.287 | 0.49 | 0.13-1.83 | |
| Node |  |  |  |  |  |  | |
| N1 | < 0.001 | 3.19 | 1.60-6.36 | 0.533 | 1.31 | 0.56-3.07 | |
| NX | 0.205 | 0.82 | 0.60-1.12 | 0.148 | 0.78 | 0.56-1.09 | |
| Stage |  |  |  |  |  |  | |
| ii | 0.507 | 1.23 | 0.66-2.28 | 0.029 | 4.47 | 1.17-17.07 | |
| iii | < 0.001 | 2.51 | 1.67-3.77 | 0.005 | 4.58 | 1.58-13.29 | |
| iv | < 0.001 | 6.30 | 4.31-9.23 | < 0.001 | 25.83 | 5.40-123.56 | |
| Grade |  |  |  |  |  |  | |
| G2 | 0.993 | 7409382.076 | 0-Inf | 0.994 | 6584112.7 | 0-Inf | |
| G3 | 0.993 | 13465086.28 | 0-Inf | 0.994 | 8761943.47 | 0-Inf | |
| G4 | 0.993 | 35125243.36 | 0-Inf | 0.994 | 9873301.98 | 0-Inf | |
| Laterality (Right) | 0.029 | 0.72 | 0.53-0.97 | 0.159 | 0.159 | 0.58-1.09 | |
