# Supplementary material for: An immune infiltration-related prognostic model of kidney renal clear cell carcinoma with two valuable markers: CAPN12 and MSC
Source: Front Oncol. 2023 Mar 21;13:1161666. doi: 10.3389/fonc.2023.1161666 (PMC10071012; doi:10.3389/fonc.2023.1161666)
Supplement: Supplementary Table 10 — Univariate and multivariate analysis regarding DSS in the prognostic model. [file Table_10.docx]

| Characteristic | P | HR | 95%CI | P | HR | | 95%CI |
| --- | --- | --- | --- | --- | --- | --- | --- |
| Univariate Multivariate | | | | | | | |
| Riskscore | < 0.001 | 40.35 | 18.00-90.47 | < 0.001 | 15.59 | 5.90-41.20 | |
| Agegroup (Young) | < 0.001 | 0.75 | 0.51-1.10 | 0.371 | 0.84 | 0.56-1.24 | |
| Gender (Male) | 0.415 | 1.19 | 0.79-1.79 | 0.617 | 1.13 | 0.71-1.79 | |
| Tumor |  |  |  |  |  |  | |
| T2 | < 0.001 | 4.59 | 2.26-9.31 | 0.002 | 0.15 | 0.04-0.49 | |
| T3 | < 0.001 | 8.52 | 4.79-15.16 | 0.003 | 0.22 | 0.08-0.60 | |
| T4 | < 0.001 | 34.15 | 14.29-81.63 | 0.017 | 0.21 | 0.06-0.75 | |
| Metastasis |  |  |  |  |  |  | |
| M1 | < 0.001 | 8.84 | 6.01-12.98 | 0.187 | 0.36 | 0.08-1.64 | |
| MX | 0.684 | 1.34 | 0.32-5.54 | 0.295 | 0.35 | 0.05-2.48 | |
| Node |  |  |  |  |  |  | |
| N1 | < 0.001 | 3.85 | 1.75-8.49 | 0.503 | 1.37 | 0.55-3.41 | |
| NX | 0.271 | 0.80 | 0.54-1.19 | 0.119 | 0.71 | 0.46-1.09 | |
| Stage |  |  |  |  |  |  | |
| ii | 0.002 | 4.42 | 1.75-11.1 | < 0.001 | 16.85 | 3.74-75.82 | |
| iii | < 0.001 | 7.62 | 3.61-16.10 | < 0.001 | 16.89 | 4.91-58.12 | |
| iv | < 0.001 | 30.48 | 15.12-61.46 | < 0.001 | 196.05 | 30.63-1254.72 | |
| Grade |  |  |  |  |  |  | |
| G2 | 0.994 | 3023550.74 | 0.13-0.26 | 0.995 | 1016012.68 | 0-Inf | |
| G3 | 0.994 | 8989273.07 | 0-Inf | 0.995 | 1693293.62 | 0-Inf | |
| G4 | 0.993 | 29183963.22 | 0-Inf | 0.995 | 1684088.09 | 0-Inf | |
| Laterality (Right) | 0.024 | 0.64 | 0.44-0.94 | 0.178 | 0.76 | 0.50-1.14 | |
