# Supplementary material for: An immune infiltration-related prognostic model of kidney renal clear cell carcinoma with two valuable markers: CAPN12 and MSC
Source: Front Oncol. 2023 Mar 21;13:1161666. doi: 10.3389/fonc.2023.1161666 (PMC10071012; doi:10.3389/fonc.2023.1161666)
Supplement: Supplementary Table 11 — Univariate and multivariate analysis regarding PFS in the prognostic model. [file Table_11.docx]

| Characteristic | P | HR | 95%CI | P | HR | | 95%CI |
| --- | --- | --- | --- | --- | --- | --- | --- |
| Univariate Multivariate | | | | | | | |
| Riskscore | < 0.001 | 15.80 | 8.07-30.93 | < 0.001 | 3.83 | 1.78-8.23 | |
| Agegroup (Young) | 0.137 | 0.79 | 0.57-1.08 | 0.115 | 0.77 | 0.55-1.07 | |
| Gender (Male) | 0.031 | 1.47 | 0.70-1.31 | 0.009 | 1.66 | 1.13-2.44 | |
| T2 | < 0.001 | 3.46 | 2.04-5.86 | 0.191 | 0.49 | 0.17-1.43 | |
| T3 | < 0.001 | 6.20 | 4.12-9.32 | 0.265 | 0.58 | 0.23-1.51 | |
| T4 | < 0.001 | 18.07 | 8.56-38.16 | 0.016 | 0.22 | 0.06-0.75 | |
| M1 | < 0.001 | 8.99 | 6.47-12.49 | 0.206 | 0.39 | 0.09-1.69 | |
| MX | 0.926 | 0.95 | 0.30-3.00 | 0.070 | 0.19 | 0.03-1.14 | |
| N1 | < 0.001 | 4.00 | 2.00-8.02 | 0.191 | 1.70 | 0.77-3.74 | |
| NX | 0.135 | 0.78 | 0.56-1.08 | 0.018 | 0.65 | 0.46-0.93 | |
| ii | 0.010 | 2.39 | 1.23-4.66 | 0.067 | 3.27 | 0.92-11.59 | |
| iii | < 0.001 | 4.66 | 2.89-7.54 | 0.003 | 5.02 | 1.71-14.80 | |
| iv | < 0.001 | 20.02 | 12.69-31.58 | < 0.001 | 61.61 | 10.59-358.51 | |
| G2 | 0.673 | 1.533375866 | 0.21-11.21 | 0.398 | 0.412 | 0.05-3.17 | |
| G3 | 0.190 | 3.742432727 | 0.52-26.95 | 0.708 | 0.68 | 0.09-5.15 | |
| G4 | 0.011 | 13.18936747 | 1.82-95.59 | 0.857 | 1.21 | 0.16-9.39 | |
| Laterality (Right) | 0.008 | 0.65 | 0.47-0.89 | 0.027 | 0.69 | 0.50-0.96 | |
